# Crystal Structure of MpPR-1i, a SCP/TAPS protein from *Moniliophthora perniciosa*, the fungus that causes Witches’ Broom Disease of Cacao

**DOI:** 10.1038/s41598-017-07887-1

**Published:** 2017-08-10

**Authors:** Renata M. Baroni, Zhipu Luo, Rabih Darwiche, Elissa M. Hudspeth, Roger Schneiter, Gonçalo A. G. Pereira, Jorge M. C. Mondego, Oluwatoyin A. Asojo

**Affiliations:** 1 Genomics and Expression Laboratory (LGE), Institute of Biology, CP 6109, 13083–862 UNICAMP, Campinas, Brazil; 2 Agronomic Institute (IAC), CP 28, CEP 13012-970, Campinas, Brazil; 3 Synchrotron Radiation Research Section, Macromolecular Crystallography Laboratory, National Cancer Institute, Argonne, Illinois 60439 USA; 4 Department of Biology, University of Fribourg, Chemin du Museé 10, 1700 Fribourg, Switzerland; 5 National School of Tropical Medicine, Baylor College of Medicine, Houston, TX 77030 USA

## Abstract

The pathogenic fungi *Moniliophthora perniciosa* causes Witches’ Broom Disease (WBD) of cacao. The structure of MpPR-1i, a protein expressed by *M*. *perniciosa* when it infects cacao, are presented. This is the first reported *de novo* structure determined by single-wavelength anomalous dispersion phasing upon soaking with selenourea. Each monomer has flexible loop regions linking the core alpha-beta-alpha sandwich topology that comprise ~50% of the structure, making it difficult to generate an accurate homology model of the protein. MpPR-1i is monomeric in solution but is packed as a high ~70% solvent content, crystallographic heptamer. The greatest conformational flexibility between monomers is found in loops exposed to the solvent channel that connect the two longest strands. MpPR-1i lacks the conserved CAP tetrad and is incapable of binding divalent cations. MpPR-1i has the ability to bind lipids, which may have roles in its infection of cacao. These lipids likely bind in the palmitate binding cavity as observed in tablysin-15, since MpPR-1i binds palmitate with comparable affinity as tablysin-15. Further studies are required to clarify the possible roles and underlying mechanisms of neutral lipid binding, as well as their effects on the pathogenesis of *M*. *perniciosa* so as to develop new interventions for WBD.

## Introduction

Cacao seeds provide chocolate, a valued treat worldwide. A major threat to cacao production is the basidiomycete fungus *Moniliophthora perniciosa* that causes the Witches’ Broom Disease (WBD) of cacao, one of the most devastating plant diseases in the Americas^1–3^. *M*. *perniciosa* is a hemiobiotrophic fungus with an atypical prolonged biotrophic stage, lasting 60 to 90 days, when it slowly grows inside the apoplast of infected plants, inducing conspicuous morphological alterations that culminate with the formation of anomalous structures called “green brooms”^1–3^. The green brooms are chlorotic and swollen shoots that result from hormonal imbalances and intense plant metabolic reprogramming induced by fungal infection^3, 4^. The necrotophic phase of WBD occurs upon death of plant tissues invaded by proliferative mycelia. The infection of cacao fruits by *M*. *perniciosa* results in swelling, abnormal ripening, and the death of the infected tissues^1–3^.

Recent gene expression analysis revealed that some *M*. *perniciosa* pathogenesis–related-1 (MpPR-1) genes are highly and specifically expressed during green broom stage of WBD, and in germinating basidiospores^3, 5^, suggesting that MpPR-1 proteins have roles on *M*. *perniciosa* infective process. MpPR-1 proteins are homologues of plant pathogenesis–related-1 (PR-1) proteins that were first identified over a century ago as important for defense against fungi and parasites^6^. PR-1 proteins are members of the eukaryotic CAP (cysteine-rich secretory protein/antigen 5/pathogenesis related-1) or SCP/TAPS (Sperm-coating protein/Tpx/antigen 5/pathogenesis related-1/Sc7) superfamily of proteins, which has been implicated in biological processes like reproduction, fungal virulence, cellular defense, and immune evasion^6–11^. Interestingly, the SCP/TAPS protein from the phytopathogenic fungus *Fusarium oxysporum* f. sp. *lycopersici*, was shown to cause disease in immune suppressed mice^12^.

SCP/TAPS proteins are characterized by a ~15 kDa cysteine-rich CAP domain, with limited sequence identity^7–10, 13–23^. While a majority of eukaryotic SCP/TAPS proteins only have one CAP domain, some parasite CAP proteins have two covalently linked CAP domains. The structure of a representative two-CAP from *Necator americanus* has been reported^14^. The CAP domain has been implicated in lipid binding and transport and at least three unique lipid binding regions have been verified in SCP/TAPS proteins^21, 24–26^. One of the lipid binding regions was identified in tablysin-15 as a hydrophobic channel that binds leukotrienes with submicro-molar affinities, that allows the protein to function as an anti-inflammatory scavenger of eicosanoids^21^. The second lipid-binding region was defined as the sterol binding caveolin-binding motif (CBM) of the yeast CAP proteins required for *in vivo* transport of cholesterol^26–28^. The third lipid binding motifs are on the surface of human GLIPR2/GAPR-1, which binds up to three phosphatidylinositol molecules^24, 25^. The three lipid binding cavities of SCP/TAPS proteins are unique and unconnected in all reported monomer structures^7–10, 13–23^.

In addition to lipid binding motifs, SCP/TAPS proteins are characterized by a large central CAP cavity as large as 1638 Å^3^ in the case of Pry1^26^. Early studies of SCP/TAPS proteins indicated that the central CAP cavity contained a tetrad of residues, two His and two Glu that bind divalent cations including Zn^2+^ and Mg^2+^ ^15, 22, 29, 30^. The tetrad was shown to be important for Zn^2+^ binding and heparin-sulfate dependent inflammatory modulation mechanisms of cobra CRISP natrin^29^. The tetrad residues are contributed by four poorly conserved CAP motifs defined by Gibbs and colleagues^22^. Additionally the CAP cavity is independent of the lipid cavities and not connected within the monomer. A crystallographic dimer is formed in the Pry1 crystal structure in which the central CAP cavity is connected to the CBM^26^. It remains unknown if this crystallographic dimer has any functional roles^26^. Furthermore, the CAP tetrad is not required for sterol transport because SmVAL4, a CAP protein lacking the tetrad, is able to effectively transport sterol *in vivo* and bind sterol *in vitro* ^31^. Additionally, mutating the tetrad did not reduce the ability of Pry1 to bind and transport sterols^27^. These studies indicate that SCP/TAPS proteins have independent lipid and cation binding functions.

Despite having a conserved alpha-beta-alpha sandwich topology, SCP/TAPS proteins are ~50% loops, which makes it difficult to predict their structures^13, 26, 31, 32^. We present in this report the structure of MpPR-1i, a SCP/TAPS protein expressed by *M*. *perniciosa* during biotrophic stage of WBD, in basidiomes, and in monokaryotic mycelia^33^. MpPR-1i has less than 25% sequence identity with any of the structures in the protein data bank, which hampered efforts at solving the structure using molecular replacement. The crystal structure of MpPR-1i was determined using selenourea (SeUrea) soaking method to solve the phase problem^34^. This is the first *de novo* structure determined by SeUrea phasing.

## Results

### Recombinant MpPR-1i

Recombinant MpPR-1i without signal peptide was produced using a pET expression system (Figures S.1 and S.2). After purification, MpPR-1i was approximately ~99% pure and migrates on a reducing Coomassie stained SDS PAGE gel at ~17 kDa (Figures S.1 and S.2). Mass spectrometry analysis of MpPR-1i reveals a mass of 16.1 kDa, which is close to the theoretical monomer molecular weight of 16.5 kDa (Figure S.3). MpPR-1i elutes from a size exclusion column as a single sharp peak of ~16.8 kDa (Figure S.4), which is consistent with the estimate from dynamic light scattering (Table S.1). Circular dichroism profile is as expected for SCP/TAPS proteins (Figure S.5).

Using TLC analysis, a neutral saturated lipid was found bound to recombinant MpPR-1i (Figure S.6). Attempts at identifying the lipid by mass spectrometry failed, likely due to experimental limitations related to their ionization of neutral lipids as was previously observed in studies of HIF-3α where the authors identified the nature of the phospholipids but were unable to identify neutral lipids^35^. Interestingly, the crystal structure of MpPR-1i did not reveal any electron density for bound lipid, which is not unusual considering the low resolution of the structure and also could result from the crystallization agents outcompeting the lipid or the conformational flexibility of the lipid. The lipid identified by TLC was usurped during recombinant production in *E*. *coli* and may not be the same lipid that MpPR-1i binds endogenously when *M*. *perniciosa* infects cacao. Future studies beyond the scope of this manuscript include identifying the major lipids secreted during this infective process and determining if MpPR-1i is capable of binding to them.

### Structure Determination

All attempts at molecular replacement failed, which was not unexpected since MpPR-1i shares less than 25% sequence identity to any known structure. Despite the large number of sulfur atoms, attempts at single wavelength anomalous phasing using S signal (S-SAD) failed. Single wavelength Se anomalous data were collected to 2.9 Å resolution after soaking a single crystal with SeUrea, and nine SeUrea binding sites were identified. Using these phases, 1225 amino acid residues corresponding to seven monomers were built into the asymmetric unit (Table 1). In the refined model six SeUrea are located at the interface of adjacent monomers, while three are relatively weak binding sites (Figure S.7). SeUrea interacts with the carboxyl group from the side chain of Gln68 and the main chain of Val122 through hydrogen bonds (Figure S.7). The structure was refined and extended to higher resolution, using a 2.43 Å native data set. Coordinates and structure factors for both models have been deposited in the Protein Data Bank under accession numbers 5V50 (native) and 5V51 (SeUrea).

**Table 1:** Statistics for data collection and model refinement.

Data Collection	MpPR-1i	MpPR-1i SeUrea
	PDB entry 5V50	PDB entry 5V51
X-ray Source	APS-22ID	APS-22ID
Detector	Rayonix MX300HS	Rayonix MX300HS
Wavelength (Å)	0.978	0.978
Space group	*P*2_1_	*P*2_1_
Cell dimensions	a = 106.3 Å, b = 84.1 Å, c = 130.9 Å	a = 108.4 Å, b = 81.9 Å, c = 130.2 Å
	α = γ = 90.00°, β = 111.4°	α = γ = 90.00°, β = 111.6°
Resolution (Å)	50.00–2.43 (2.52–2.43)	50.00–2.90 (3.00–2.90)
Number of total reflections	622,438	341,996
Number of unique reflections	80,587 (7,964)	46,260 (4,495)
^†^ *R*_*merge*_ (%)	9.3 (142.8)	11.7 (112.2)
*I*/*σ*(*I*)	18.9 (1.6)	15.8 (1.4)
Completeness (%)	99.4 (98.9)	99.6 (99.8)
^†^Redundancy	7.7 (7.8)	7.4 (6.2)
CC_1/2_	0.977 (0.891)	0.975 (0.867)
Wilson B-factor (Å^2^)	57.1	54.6
**Refinement (REFMAC5)**		
Resolution (Å^2^)	45.09–2.43 (2.49–2.43)	45.07–2.92 (2.99–2.92)
^a^ *R*_*work*_	0.201 (0.444)	0.187
^b^ *R*_*Free*_	0.243 (0.467)	0.228
r.m.s. deviation bond length (Å)	0.014	0.010
r.m.s. deviation bond angles (°)	1.435	1.343
MolProbity analysis		
Ramachandran outliers	0.65%	0.77%
Ramachandran favored	94.47%	94.60%
No. of non-H protein atoms	7433	7315
No. of water molecules	76	0
Ions and ligands	0	9 SeUrea
Correlation coefficient *F*_*o*_-*F*_*c*_	0.965 (0.949)	0.935 (0.904)
Average B-factors (Å^2^)	86.9	72.1

### Overall Structure of MpPR-1i

**Figure 1:**
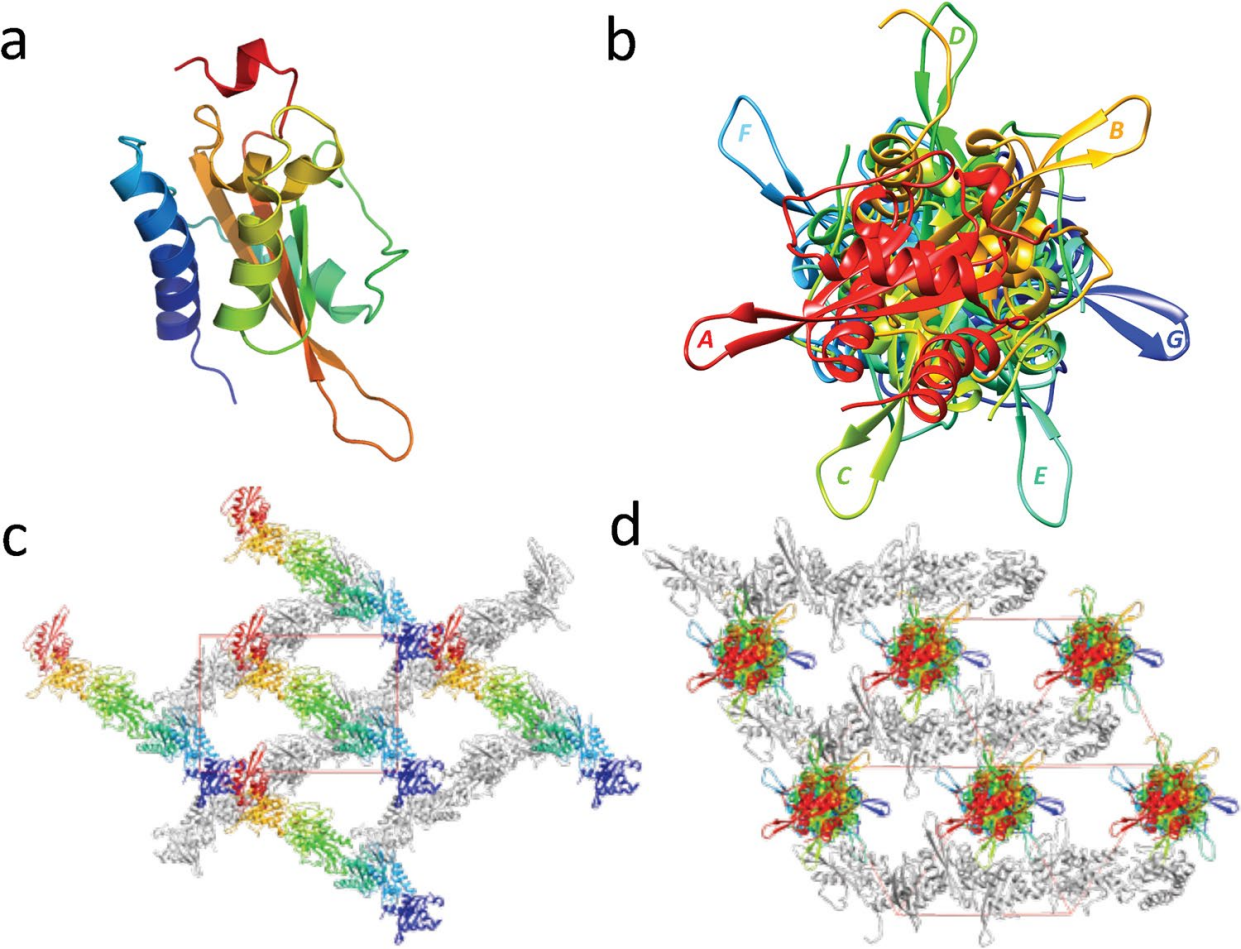
Crystal structure and packing of MpPR-1i. (**a**) Cartoon of monomer A colored in rainbow from blue (N-ter) to red (C-ter). (**b**) MpPR-1i monomers in asymmetric unit viewed along the diagonal of unit cell shows a pseudo-seven fold screw axis; each monomer is labeled along the direction of (110) to (011) as A, B, C, D, E, F, and G respectively. (**c**) Crystal packing presented along *a* cell dimension. Monomers in the top layer are colored as in Fig. 1b, while the bottom layer monomers are shown in gray. The large solvent channel formed by crystal packing is also visible. (**d**) Crystal packing viewed along the cell diagonal.

Each monomer of MpPR-1i has a conserved alpha-beta-alpha sandwich topology made up of 3 β strands sandwiched between two helical domains, connected by loops (Fig. 1a). One of these loops connects the two longest β strands, extends out from the core structure, and is exposed to the solvent channel in crystal. There are seven monomers in the asymmetric unit, which form a pseudo seven fold screw axis when viewed along the diagonal of the cell (Fig. 1b,c and d). The MpPR-1i crystal has very high solvent content, ~70%, which is clearly demonstrated by the solvent channel in the crystal packing viewed along *a* cell dimension (Fig. 1c).

**Figure 2:**
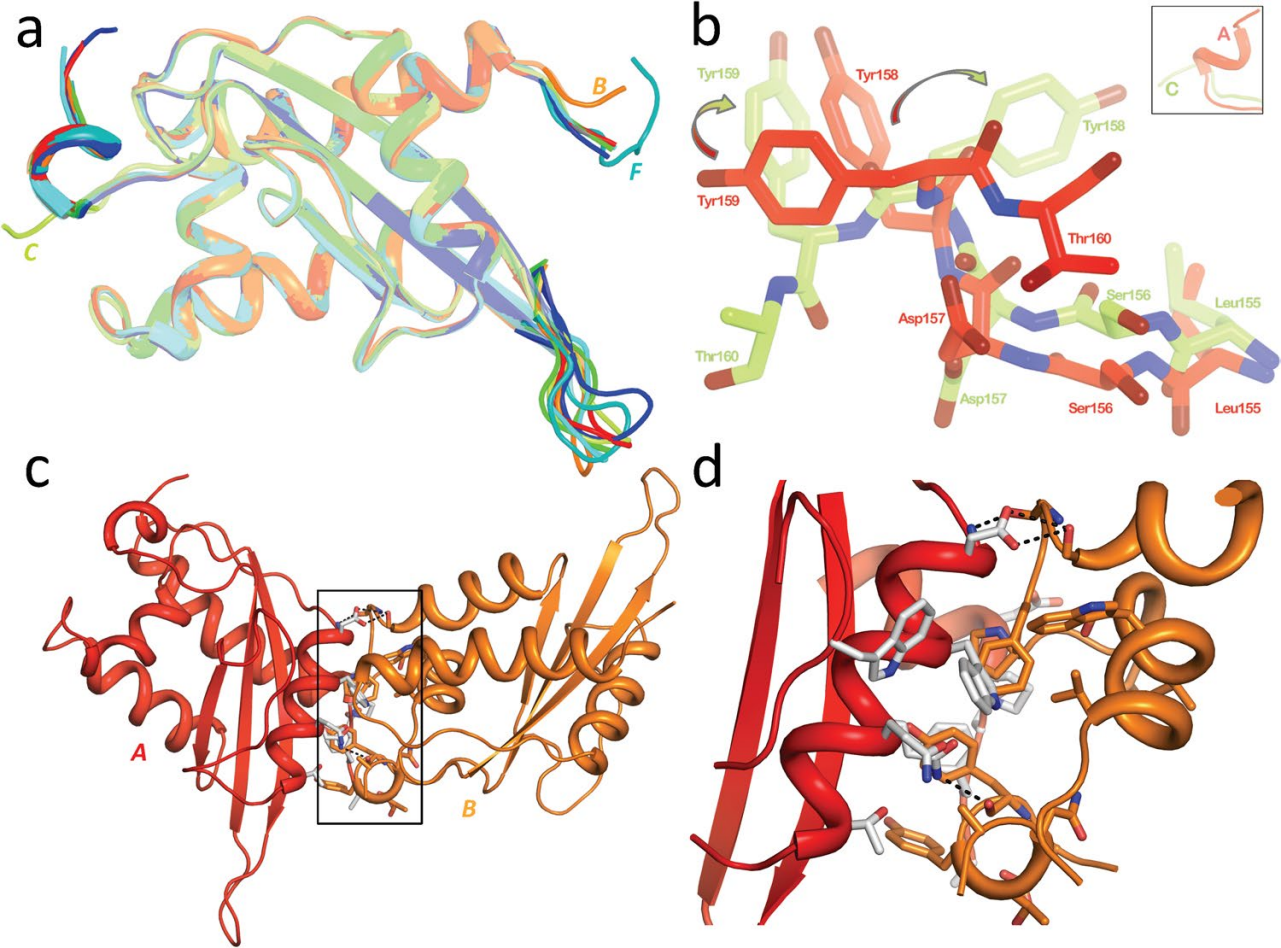
Structure similarity and intermolecular interaction of MpPR-1i. (**a**) Superposition of all seven monomers in asymmetric unit reveals that loop regions at termini and between longest β-sheet as the most variable parts. (**b**) Comparison of C-termini of monomers A and C. The insert reveals that monomer C and A have the overall opposite orientation starting from the peptide bond between Asp157, Tyr158, and Tyr159 in molecule C rotate ~90° clockwise compared to the equivalent residues in molecule A indicated by arrow. (**c**) Global view of intermolecular interaction of MpPR-1i. (**d**) Network of interactions between monomer A and B. The carbon atoms are colored as gray in molecule A and orange in molecule B. Oxygen atoms are shown as red and nitrogen atoms are presented as blue. The hydrogen bonds are shown as black dash.

The main chains of the MpPR-1i monomers are very similar with rmsd ranging between 0.19 Å to 0.27 Å. The most variable regions between the monomers are loop regions, notably the solvent exposed loop connecting the two longest β-stands, as well as the N- and C-termini loops (Fig. 2a). The amino termini of 6 monomers have the same orientation, while one (labeled monomer B) has a different orientation (Fig. 2a). While six monomers have conserved C-ter loops, the main and side chain residues starting from Leu155 in the carboxyl terminus of one (labeled monomer C) are flipped in an opposite conformation from the other monomers. Notably residues Tyr158 and Tyr 159 in monomer C are oriented 90° away from what is observed in the other monomers (Fig. 2a and b). The interface between adjacent monomers appears to be crucial for crystal packing and have a buried surface area of ~800 Å^2^ per monomer. None of the intermolecular contacts between monomers have more than 8 hydrogen bonds and the majority of the residues at the monomer interface are hydrophobic residues as illustrated by the interface between monomers A and B (Fig. 2c and d).

### Central CAP cavity

**Figure 3:**
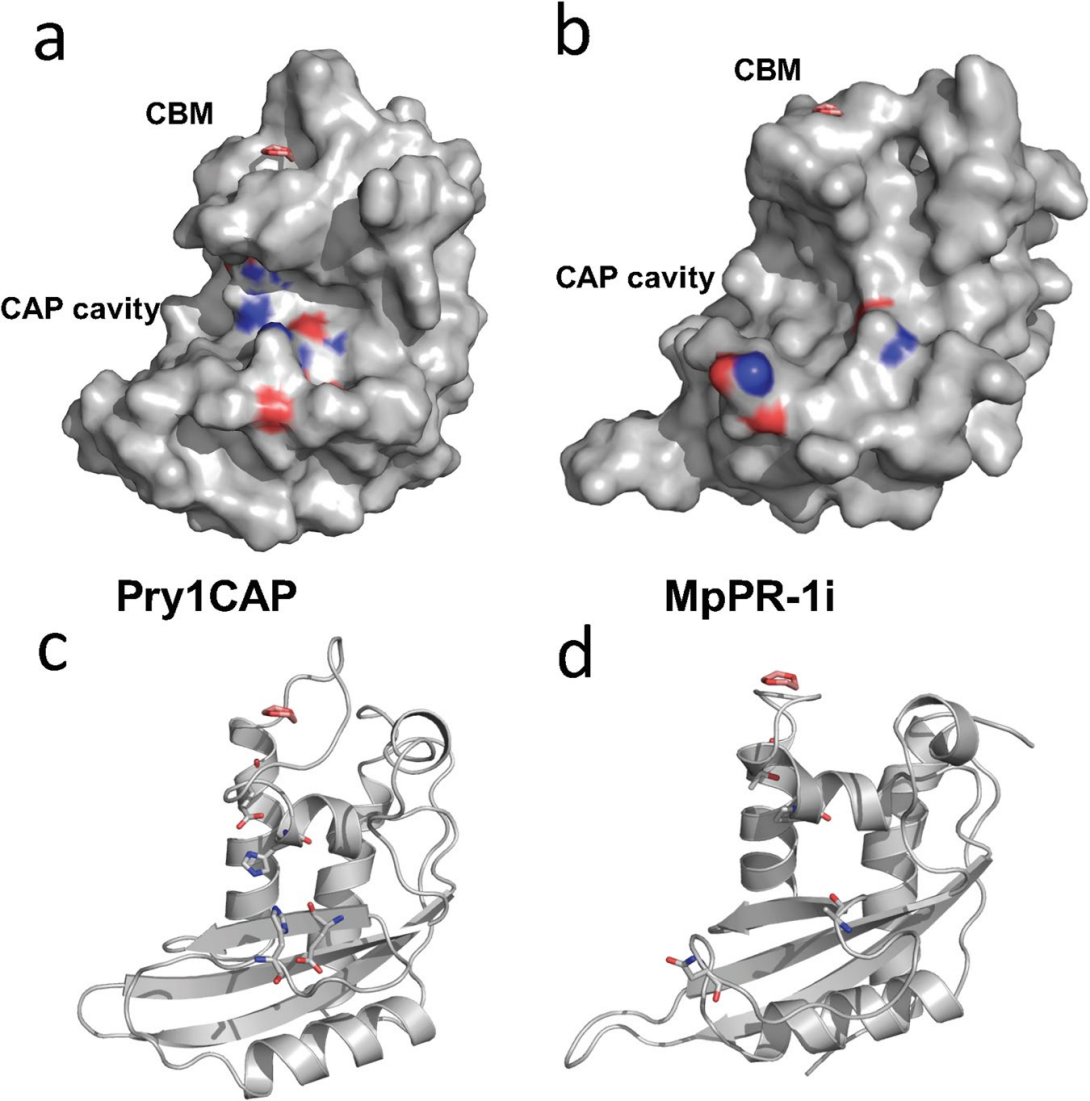
Comparison of CAP cavity of Pry1CAP and MpPR-1i. (**a**) Surface diagram of Pry1CAP and (**b**) MpPR-1i reveal central CAP cavity and Caveolin-binding motif (CBM) containing dioxane (orange stick), ribbon diagram of equivalent view of (**c**) Pry1CAP and (**d**) MpPR-1i monomer showing CBM containing dioxane (orange stick) and CAP tetrad (stick).

**Figure 4:**
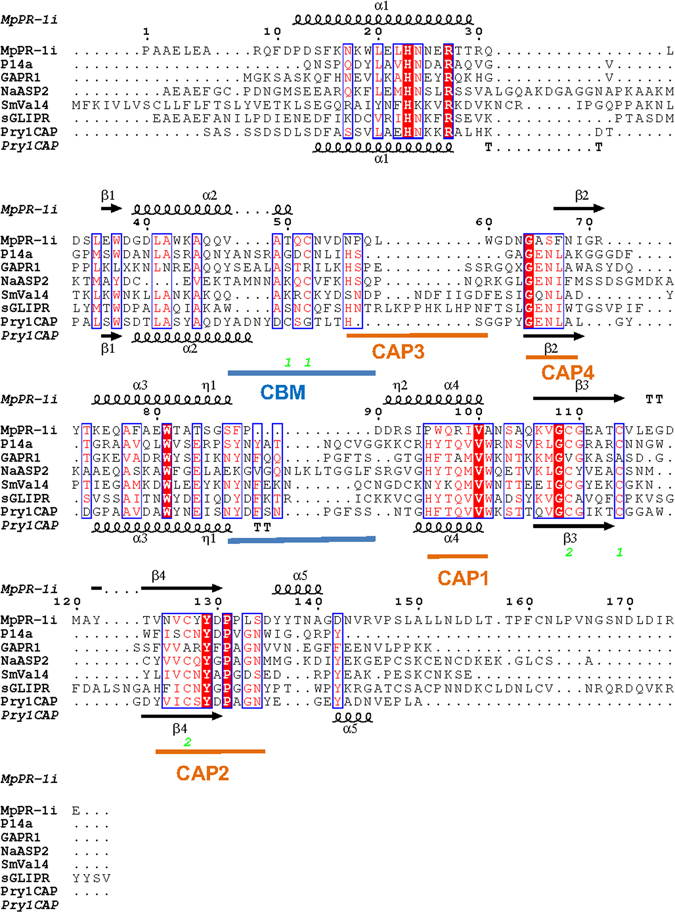
SCP/TAPS protein motifs. Structural features of MpPR-1i and primary sequence alignment with SCP/TAPS proteins that are most structurally similar. This figure was generated with ESPript^56^. The different secondary structure elements shown are alpha helices as large squiggles labelled (α), 3_10_-helices as small squiggles labelled (η), beta strands as arrows (β), and beta turns (TT). Identical residues are shown in white on red background, and conserved residues in red. The locations of the cysteine residues involved in disulfide bonds are numbered in green. CAP motifs are highlighted in orange, and caveolin-binding motif is indicated in blue. The SCP/TAPS structures with pdb accension codes in parenthesis are *Na*-ASP-2 (1u53), SmVAL4 (4p27), PI14a (1cfe), GAPR-1 (1smb), Pry1CAP (5ete), and sGLIPR1 (PDB entry 3q2r).

Like other reported SCP/TAPs protein structures, MpPR-1i has a large central CAP cavity (Fig. 3a,b)^13, 15, 22, 29, 36–38^. The volume of the CAP cavity of MpPR-1i is 1334.39, Å^3^ which is comparable to the large size previously observed in Pry1 at 1638 Å^3^. In many CAP proteins, the central CAP cavity contains a tetrad formed by residues from four signature CAP motifs: His from CAP1, Glu from CAP2, His from CAP3, and Glu from CAP4. These tetrad residues bind divalent cations including Zn^2+^ and Mg^2+^ (Fig. 3c,d)^13, 15, 16, 21, 22, 24, 29, 30, 39^. MpPR-1i, like SmVAL4, lacks the tetrad that binds divalent cations in other SCP/TAPS proteins^31^ (Figs 3 and 4). This explains why MpPR-1i does not bind Zn^2+^ used in the crystallization solution. It remains unknown why some SCP/TAPS proteins have the conserved tetrad while others do not; however the absence of the tetrad in MpPR-1i means it lacks the ability to bind divalent cations and will not be involved in heparin-sulfate dependent inflammatory modulation mechanisms like natrin^29^.

### Lipid binding by MpPR-1i

**Figure 5:**
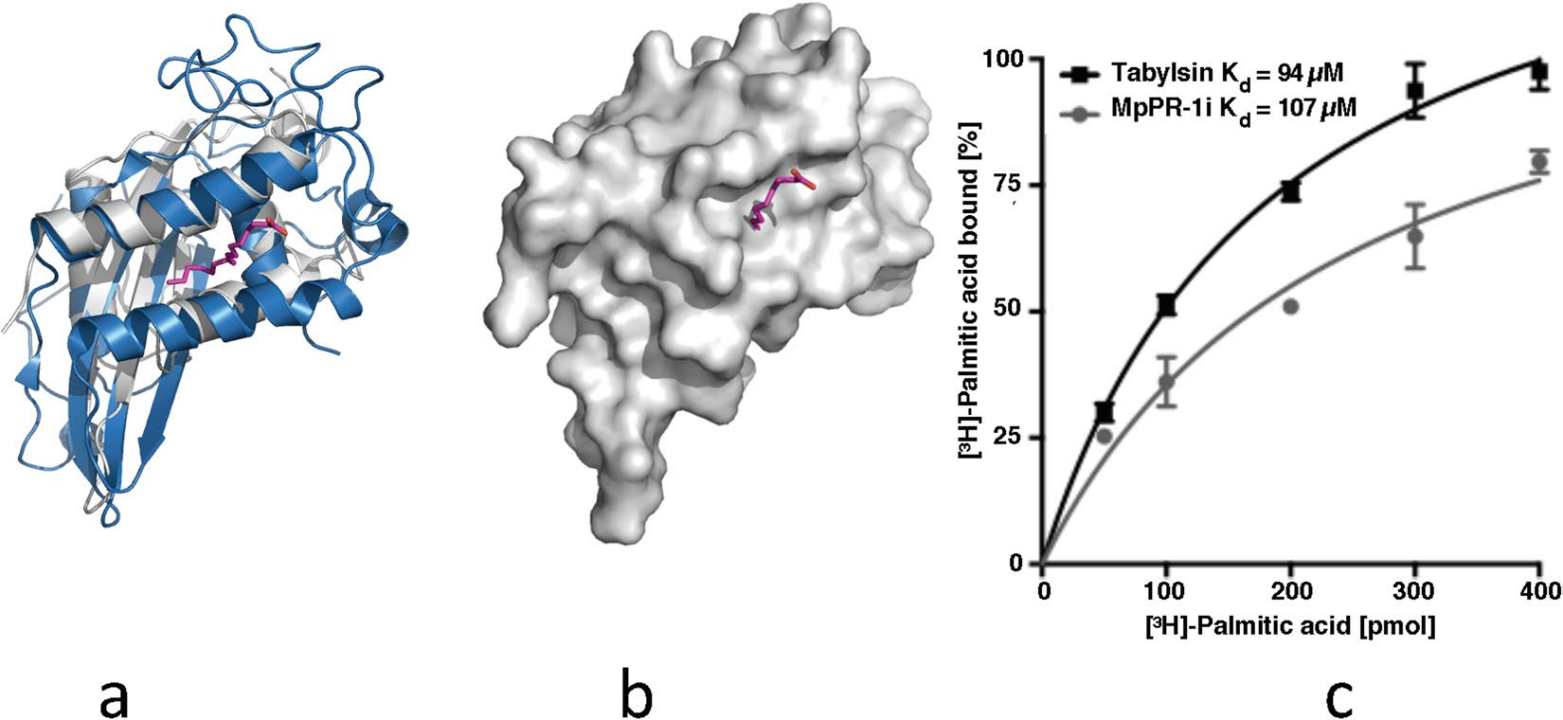
MpPR-1i has a large lipid binding site like tablysin-15. (**a**) Superpositioning of MpPR-1i (gray) with tablysin-15 (blue) reveals similar sized palmitate (magenta) binding cavity. (**b**) Surface diagram reveals that the cavity is large enough to accommodate palmitate. (**c**) *In vitro* binding affinity of palmitate to MpPR-1i.

Since TLC analysis shows that MpPR-1i binds to a neutral saturated lipid, the structure was analyzed to see if it has any of the known lipid binding cavities. Structural comparisons with tablysin-15, a palmitate binding CAP protein, showed that MpPR-1i has a similar cavity sufficiently large and open to accommodate palmitate or similar lipids (Fig. 5a and b). The binding affinity of MpPR-1i for palmitate was determined using our established *in vitro* lipid-binding assay^27^ and this analysis showed that MpPR-1i binds palmitic acid. The measured estimated equilibrium constant for MpPR-1i is K_d_ 107 μM, which is comparable to that of tablysin-15 with a K_d_ of 94 μM^36^ (Fig. 5c).

## Discussion

### Selenourea phasing

All attempts at molecular replacement failed regardless of search model used so we tried phasing by anomalous diffraction. Although the crystallization condition contains zinc acetate, no anomalous signal for Zn^2+^ ions was observed in any of the data sets, which was expected since MpPR-1i lacks the CAP tetrad. SeUrea soaking provided sufficient anomalous signal to phase the crystal structure of MpPR-1i. The low resolution SAD data at 2.9 Å has enough anomalous signal to locate the Se atoms, and enough reflections to build the whole model even without native data. This approach enables the use of SeUrea quantitatively and can be adapted for phasing other structures. As previously discussed, SeUrea does not form a stable aqueous solution, so a reducing agent like sodium sulfite (Na_2_SO_3_) or TCEP is added to slow down the oxidation of SeUrea^34^. The stability of SeUrea was improved by using a higher concentration of Na_2_SO_3_ to prepare the 1 M SeUrea/Na_2_SO_3_ solution, allowing the stock solution to be stored at −20 °C for several months.

### Oligomerization of MpPR-1i

MpPR-1i forms a unique crystallographic heptamer, which likely does not have any functional relevance as MpPR-1i forms monomers in solution. Evidence supporting the monomer includes DLS revealing a MW of ~20 kDa, the absence of dimerization peaks in MS, the similar molecular mass of ~17 kDa on both reduced and non-reduced gels, and the protein elution off a sizing column as a sharp peak with a molecular mass of ~17 kDa. The formation of both monomers and dimers has been previously reported in other SCP/TAPS. While some like Na-ASP-2, GLIPR-1, and Pry1 form dimers in solution, others like SmVAL-4 form monomers^13, 15, 26, 31^. Interestingly, none of the dimers formed within the heptamer are similar to the packing of the two-CAP Na-ASP-1 or to the dimer in Pry1 that connect the CAP cavity^14, 26^. While the formation of the crystallographic heptamer has no apparent functional relevance, it explains the failure of phasing by S-SAD, because the heptamer only has 42S atoms out of 18,732 total atoms, which gives weak anomalous S signal compared to the strong Se signal from SeUrea soaking.

### Comparison of MpPR-1i with other SCP/TAPS proteins

Using PDBFold, the most similar structures to MpPR-1i were identified as the apo structure of human Golgi-associated PR-1 protein GAPR-1^16, 24^, Pry1 from yeast^26^, SmVAL4 from *Schistosoma mansoni* ^31^, the NMR structure of a plant P14a^17^, and the structures of human glioma pathogenesis related protein (sGLIPR1)^15^. MpPR-1i shares 19.4%, 24.2%, 20.8%, 24.3% and 20.2% sequence identity with these proteins respectively. While the core alpha-beta-alpha sandwich topology is conserved, MpPR-1i has different loop regions as well as helix and strand lengths compared to the other structures (Fig. 4). The regions of greatest flexibility have been implicated in ligand binding and make up ~40% of the structure. Interestingly, the caveolin binding motif (CBM) loop, which has been implicated in cholesterol binding in Pry1, is significantly shorter in MpPR-1i than in other CAP proteins (Fig. 4). The shortened length of the CBM loop significantly reduces the size of the sterol binding cavity, rendering it barely large enough to accommodate dioxane and definitely too small to accommodate cholesterol (Fig. 3). Thus structural data strongly suggests that MpPR-1i will lack the ability to bind cholesterol. *In vivo* and *in vitro* analyses of the implications of the small CBM on sterol binding by MpPR-1i are currently being investigated and will be published elsewhere.

### Lipid binding function of MpPR-1i

*MpPR-1i* gene expression was detected in monokaryotic mycelia, basidiomata, and especially in the green broom stage of the disease^33^, which suggests participation in fungal pathogenesis. The observation that MpPR-1i binds to a neutral lipid suggests that it can accommodate fatty acids in its large open palmitate binding cavity between α-helices 1 and 4 (Fig. 6a and b) as observed in SmVal4 and tablysin-15^31, 38^. Tablysin-15 is a protein present in the saliva of the horsefly *Tabanus yao*, which scavanges cysteinyl leukotriene, an eicosanoid lipid that promotes inflammatory response^38^. During plant infection, lipolytic enzymes target host cellular membranes, releasing free fatty acids, such as oxylipins, that have roles in plant immunity^40^. Indeed, the binding affinity measured in our established *in vitro* lipid binding assay was comparable to that previously observed for tablysin-15^28^. Therefore, MpPR-1i could act similarly to tablysin-15, sequestering lipids that potentiate plant defense response. Further studies are needed to determine the binding of MpPR-1i to free fatty acids that are important in plant immunity.

## Conclusions

The structure of MpPR-1i was determined by SeUrea phasing. This is the first *de novo* structure determined using this phasing technique and reveals the applicability of this method to a new structure with >70% solvent content. MpPR-1i is a compact CAP protein that is a monomer in solution but is packed as a high solvent content crystallographic heptamer. The loops connecting the two longest strands are exposed to the solvent channel and exhibit the largest inter-monomer conformational flexibility. MpPR-1i retains the palmitate binding cavity while the sterol binding CBM cavity is smaller than previously observed in other SCP/TAPS proteins. Future studies include assessing the mechanisms of lipid binding by MpPR-1i.

## Methods

### Recombinant protein expression and purification of MpPR-1i

MpPR-1i coding sequence, without signal peptide, was amplified from cDNA from WBD’s green broom stage. MpPR-1i was subcloned into pGEMT-Easy Promega, and then cloned into a modified version of pET SUMO (Invitrogen, Carlsbad, USA), which was transformed into *E*. *coli* Shuffle strain (New England Biolabs USA). The transformed cells were grown in LB medium containing kanamycin (50 µg/ml) under agitation (200 rpm) at 30 °C overnight. The protein expression was induced by the addition of IPTG (0.2 mM) after bacterial suspension achieves an optical density (OD_600_) of 0.8. The cells were incubated for 16 h at 18 °C with shaking (200 rpm), harvested by centrifugation, and subjected to chemical cell lysis and 1 cycle of freeze thaw using a solution containing 50 mM Tris-HCl (pH 8.5), 150 mM NaCl, 10% glycerol, lysozyme (150 mg/L), deoxycholic acid (40 mg/L), and DNAse I (1.25 mg/L). Supernatant was clarified by centrifugation to remove insoluble protein and cellular debris. His-tagged-MpPR-1i was purified by immobilized metal ion affinity chromatography (IMAC) using Co^2+^-charged TALON resin equilibrated with Tris-HCl (pH 8.5) and 150 mM NaCl. After extensive washing with this solution, protease ULP-1 was added for removing His-SUMO tag. After proteolytic cleavage, un-tagged MpPR-1i was eluted from the resin further purified by gel filtration using a Superdex 75 HR 16/60 column (GE) equilibrated in 50 mM Tris-HCl, pH 8.5, and 150 mM NaCl. The resulting protein was concentrated using an Amicon Ultra-15 Centrifugal Filter Unit 10 kDa membrane (Millipore, Billerica, MA, USA). Protein concentration was estimated based on UV absorbance at 280 nm, using extinction coefficient calculated from the primary sequence in the ExPASy ProtParam tool. Protein samples were lyophilized for long-term storage. More details about protein purity and purification are shown in Supplementary Methods (Figure S.1).

### Circular Dichroism

CD spectra were recorded on a spectropolarimeter (J810, JASCO) at 20 °C. CD spectra were acquired using quartz cells (path length 0.1 cm) at a protein concentration of 10 µM in 50 mM Tris-HCl pH 8.5 and 150 mM NaCl. Three trials were performed on scanning from 190 to 260 nm. Deconvulation of CD spectra were performed using DichroWeb program^41, 42^.

### Dynamic light scattering analyses

Dynamic light scattering (DLS) analyses were performed on a DynaPro Wyat DynaPRO 99-E (Wyatt Technology Corp). The experiments were conducted with an acquisition time of 10 s at 25 °C.

### Size exclusion chromatography (SEC)

SEC was performed using a Shimadzu Prominence ultra-fast liquid chromatography system (UFLC) with photo-diode array detector and a 3μm Yarra SEC-2000 (300 mm × 7.8 mm) analytical column (Phenomenex). The mobile phase was 50 mM Bis-Tris Propane pH 7.0, and the flow rate was 0.5 mL/min at room temperature. 50 µL of 1 mg/ml MpPR-1i in 50 mM Bis-Tris Propane pH 7.0 was injected on the column. Similar results were observed using 50 mM Tris-HCl pH 8.5 or PBS pH 7.4 as the mobile phase.

### Delipidation of MpPR-1i and TLC analyses

Lipids were extracted from purified protein (~1.5 mg/mL) using 1 mL of a solution containing Methyl tert-butyl ether (MTBE): methanol: water (3:1:1), previously cooled (−15 °C). Samples were shaken during 30 min at 4 °C, followed by incubation at an ice bath under ultrassonication during 10 min. Subsequently, 650 µL of a solution of methanol:water (1:3) was vigorously shaken then centrifuged at 13000 × g at 4 °C for 5 min. Upper phase was transferred to a clean tube that was dried through lyophilization. Samples were separated by thin-layer chromatography on silica gel 60 plates (TLC; Merck, Darmstadt, Germany) using two different solvent systems: cyclohexane: ethyl acetate (4:1) for unsaturated neutral lipids, and chloroform: methanol: water (75:25:2.5) for saturated neutral lipids and phospholipids, the latter being revealed by Dittmer-lester reagent. TLCs were air-dried, scanned and photographed.

### Mass Spectrometry

Lyophilized protein was reconstituted by addition of water and 5% acetonitrile prior to mass spectrometry (MS) analysis using an Impact II QTOF mass spectrometer (Bruker Daltonics), equipped with a Qtof Control and Electrospray source. MS spectra were acquired in positive ion mode using water, 5% acetonitrile, and 0.1% formic acid. Instrument parameters were set as follows: nebulizer gas (Nitrogen) pressure, 2 Bar; Capillary voltage, 4.500 V; ion source temperature, 180 °C; dry gas flow, 9 L min-1; spectra rate acquisition between m/z 300–2000.

### Crystallization and selenourea soaking

Lyophilized protein was reconstituted by addition of water prior to crystallization. Crystallization was manually optimized by hanging-drop method at room temperature. The best crystals were obtained by mixing 1 μL of 13 mg/mL MpPR-1i in 0.1 mM Tris-HCl pH 8.5 buffer, with an equal volume of well solution (2.8 M sodium formate, 70 mM Bis-Tris propane pH 7.0, and 21 mM zinc acetate). The well solution supplemented with 20% (v/v) MPD was used as cryoprotectant. Native crystals were transferred into cryoprotectant for a few seconds and vitrified in liquid nitrogen. To generate selenourea (SeUrea) derivative, a 1 M SeUrea/Na_2_SO_3_ stock solution was prepared by dissolving SeUrea into 1 M sodium sulfite (Na_2_SO_3_) and stored at −20 °C. Each MpPR-1i crystal was transferred from mother liquor into a mixture of 1.8 μL cryoprotectant and 0.2 μL of 1 M SeUrea/Na_2_SO_3_ solution. After 10 min soaking, derived crystals were vitrified in liquid nitrogen prior to data collection.

### *In vitro* palmitate binding assay

The radioligand binding assay was performed as described previously^43, 44^. Purified protein (100 pmol) in binding buffer (20 mM Tris, pH 7.5, 30 mM NaCl, 0.05% Triton X-100) was incubated with [^3^H]-palmitic acid (100–400 pmol) for 1 h at 30 °C. Protein was removed from unbound ligand by adsorption to Q-sepharose beads (GE healthcare, USA), the beads were washed, and the protein-bound radioligand was quantified by scintillation counting. To determine non-specific binding, the binding assay was performed without the addition of the protein.

### Data Collection and Structure Determination

Synchrotron X-ray diffraction data were collected at wavelength of 0.978 Å on Southeast Regional Collaborative Access Team (SER-CAT) 22-ID beam-line at the Advanced Photon Source, Argonne National Laboratory, USA. Data sets were processed with HKL2000^45^ in space group *P*2_1_ with the “auto-correction” option turned during scaling. The best SeUrea soaked crystals diffract to 2.9 Å, while the best native crystals diffract to 2.43 Å. Attempts to solve the crystal structure of MpPR-1i by molecular replacement by submitting both data to BALBES online server failed^46^. Parallel attempts at phasing using multiple MR search models, truncated CAP proteins, and polyalanine models^13–15^ with PHASER^47, 48^ were also unsuccessful. The phenix.anomalous signal in PHENIX package was used to estimate the correlation coefficient for anomalous data set processed without merging Friedel pairs^49, 50^. Correlation coefficient for anomalous data set (CC_ano_) at different resolution is shown in Figure S.8. SHELXD was used to find the sub-structure of the anomalous data and identified six Se^51^; however, attempts to build the polyalanine model even with relatively higher resolution native data using SHELXE failed. After switching to Phenix.Autosol for phasing and model building with Phenix.Autobuild, an initial model with *R* = 0.37 and *R*_free_ = 0.41 was obtained, indicating that the correct solution was found^52^. Buccaneer was adopted for further model building which resulted in an 88% complete model with *R* = 0.29 and 984 residues assigned into seven chains. The highest quality single chain was extracted and used as the molecular replacement model in PHASER^53^ to generate a more complete model. The SeUrea binding sites were cross validated by anomalous difference map and the heavy-atom sites found by Phenix.Autosol, then incorporated into model by Coot^53^. Thereafter, the structure was iteratively manually adjusted in Coot and refined using REFMAC5^54, 55^ and PHENIX^52^. The occupancies of SeUrea molecules were also refined. Data collection and structure refinement statistics are listed in Table 1.

## Electronic supplementary material

Supplementary information
