# Supplementary material for: Crystal Structure of MpPR-1i, a SCP/TAPS protein from Moniliophthora perniciosa, the fungus that causes Witches’ Broom Disease of Cacao
Source: Sci Rep. 2017 Aug 10;7:7818. doi: 10.1038/s41598-017-07887-1 (PMC5552782; doi:10.1038/s41598-017-07887-1)
Supplement: Supplementary file 1 — Supplementary information [file 41598_2017_7887_MOESM1_ESM.doc]

**Supplementary Information for Crystal Structure of MpPR-1i, a SCP/TAPS protein from *Moniliophthora perniciosa*, the fungus that causes Witches' Broom Disease of Cacao.**

Renata M. Baroni^1,2a^, Zhipu Luo^3a^, Rabih Darwiche^4^, Elissa M. Hudspeth^5^, Roger Schneiter^4^, Gonçalo A. G. Pereira^1^, Jorge M.C. Mondego^2*^, and Oluwatoyin A. Asojo^5*^

**^a^** Co-first authors; ^*^ Corresponding authors

^1^ Genomics and Expression Laboratory (LGE), Institute of Biology, CP 6109, 13083-862 UNICAMP, Campinas, Brazil

^2^ Agronomic Institute (IAC), CP 28, CEP 13012-970, Campinas, Brazil

^3^ Synchrotron Radiation Research Section, Macromolecular Crystallography Laboratory, National Cancer Institute, Argonne, Illinois 60439, USA

^4^Department of Biology, University of Fribourg, Chemin du Museé 10, 1700 Fribourg, Switzerland

^5^ National School of Tropical Medicine, Baylor College of Medicine Houston TX 77030 USA

*Running Title*: Structure of MpPR-1i

Keywords: sperm coating protein (SCP); TAPs [testis specific proteins (Tpx) / antigen 5 (Ag5) / pathogenesis related-1 (PR-1) / Sc7]; CAP [cysteine-rich secretory protein (CRISP) / antigen 5 / pathogenesis related-1 (PR-1)]; *cacao*; selenourea phasing; lipid binding; SCP/TAPS (Sperm-coating protein / Tpx / antigen 5 / pathogenesis related-1 / Sc7)

Correspondence to: asojo@bcm.edu (OAA) and jmcmondego@iac.sp.gov.br (JMCM)

**S.1) Purification of MpPR-1i**


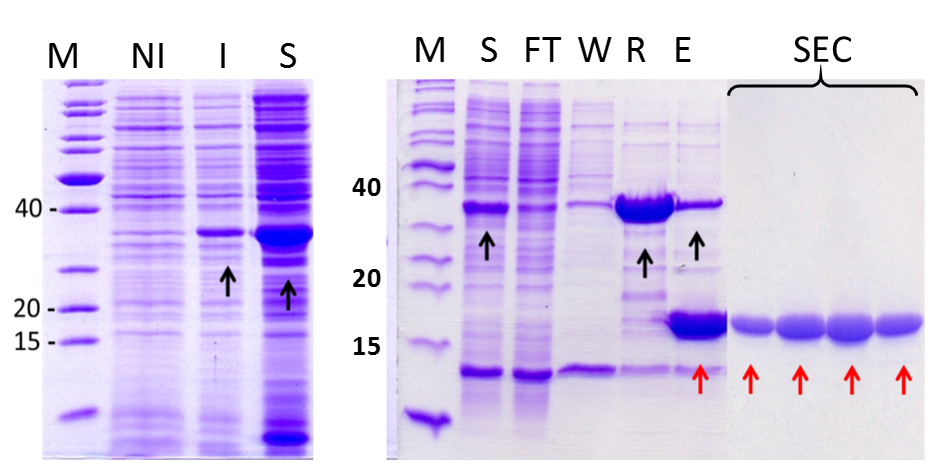


Figure S.1: Expression and purification of MpPR-1i. *Left panel*: SDS-PAGE (15% w/v) analysis of protein induction (0.2 mM IPTG). NI: non-induced, I: Induced, S: Soluble fraction. *Right panel*: SDS-PAGE (15% w/v) analysis of IMAC and SUMO-MpPR-1i cleavage. S: Soluble fraction, FT: IMAC Flow-through, W: Resin wash, R: IMAC resin; E: Elution after ULP-1 cleavage; SEC: Samples eluted from Size Exclusion Chromatography. Black Arrows: SUMO-MpPR-1i (30 kDa), Red Arrows: MpPR-1i (between 15 and 20 kDa).


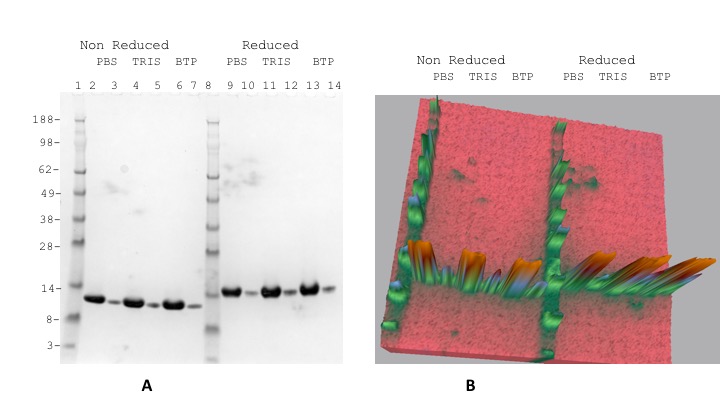


Figure S2: A) SDS-page gel showing purity of MpPR-1i in different buffers, visualized by Coomassie blue staining. Electrophoretic mobility on the reduced gel is consistent with the theoretical molecular mass of ~16.5. B) 3D gel of Fig S.2A visualized by false color. The storage buffers are 50mM Tris pH 8 (TRIS), PBS pH 7.4, and 50mM Bis tris propane pH 7 (BTP). 9μg of protein was loaded in the more concentrated lanes and 3μg of protein was loaded in the lower concentrated lanes.

**S.2) Dynamic light scattering**

Table S.1: Dynamic light scattering analyses of MpPR-1i.

| **Protein** | **Theoretical molecular weight (KDa)** | **%Pd**  **(polydispersity)** | **DLS estimated molecular weight (KDa)** | **%Int** | **% weight** |
| --- | --- | --- | --- | --- | --- |
| **MpPR-1i unique Peak** | 16.5 | 7.8 | 20 | 96.3 | 100.0 |

The data indicates that MpPR-1i is a monomer since estimated DLS weight is compatible to theoretical molecular weight. The difference in molecular weight may be due to the lipids that MpPR-1i binds during its production.

**S.3) Mass Spectrometry**

**
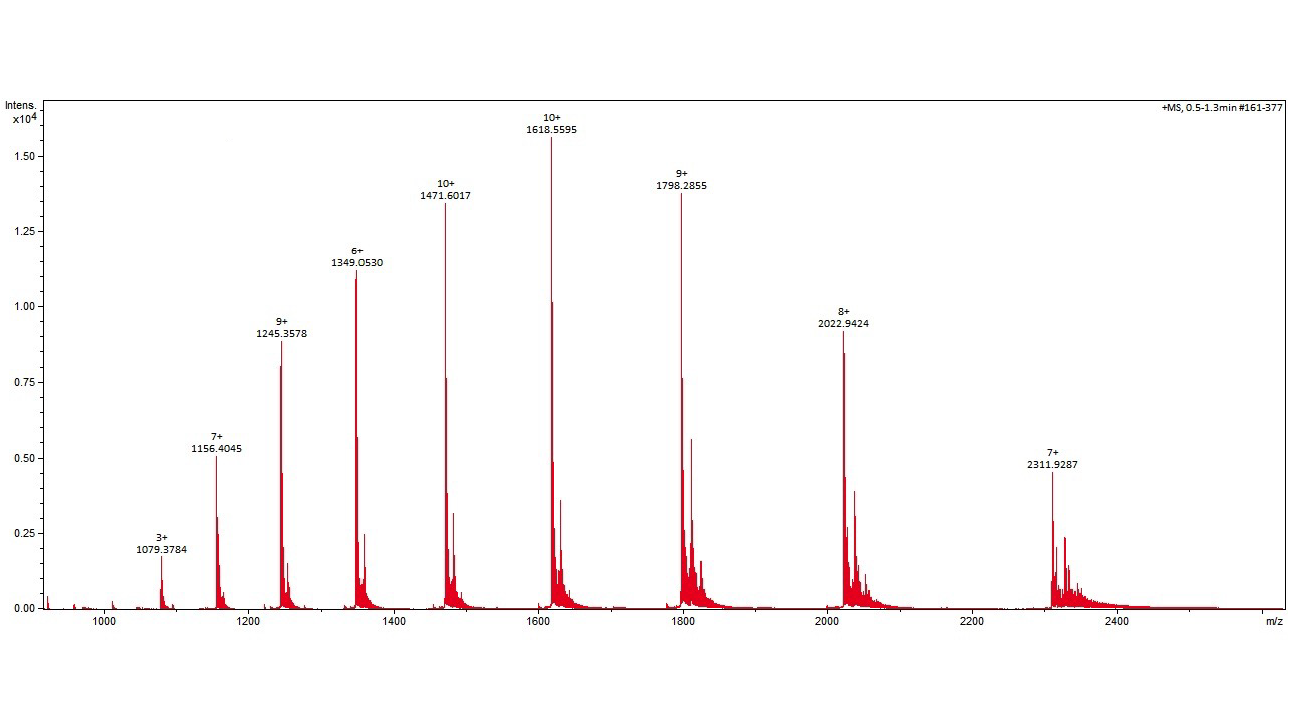
**

Figure S.3. Electrospray ionization mass spectra of intact MpPR1-i from *M. perniciosa* reveals a protein mass of 16.1 kDa, which is consistent with the theoretical mass of 16.5 kDa.

**S.4) Size exclusion chromatography**

The molecular mass of the eluted peak is determined using a calibration curve based on known standards, not just by visualization. First we calculated Kd for the standard peaks and for the sample peak and then we plotted Log MW versus Kd to get calibration curve.

Kd (distribution coefficient) = (Ve - Vo) / (Vt - Vo)

Ve = elution volume of sample

Vo = void volume (blue dextran)

Vt = total column volume (3.14*radius^2)

**
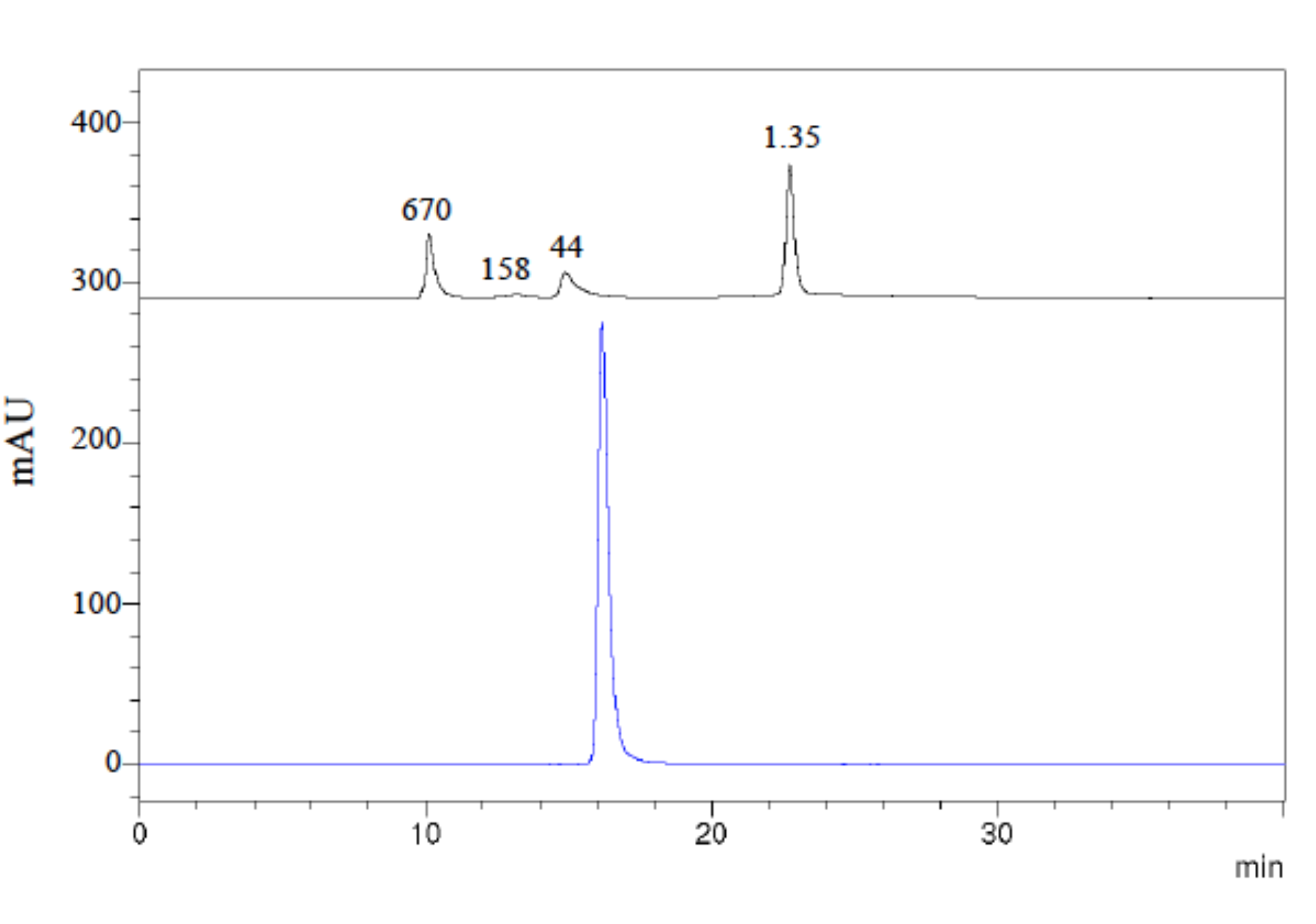
**

Figure S.4. MpPR-1i elutes as an ~16.8 kDa monomer from a size exclusion column.

**S.5) Circular dichroism analysis**


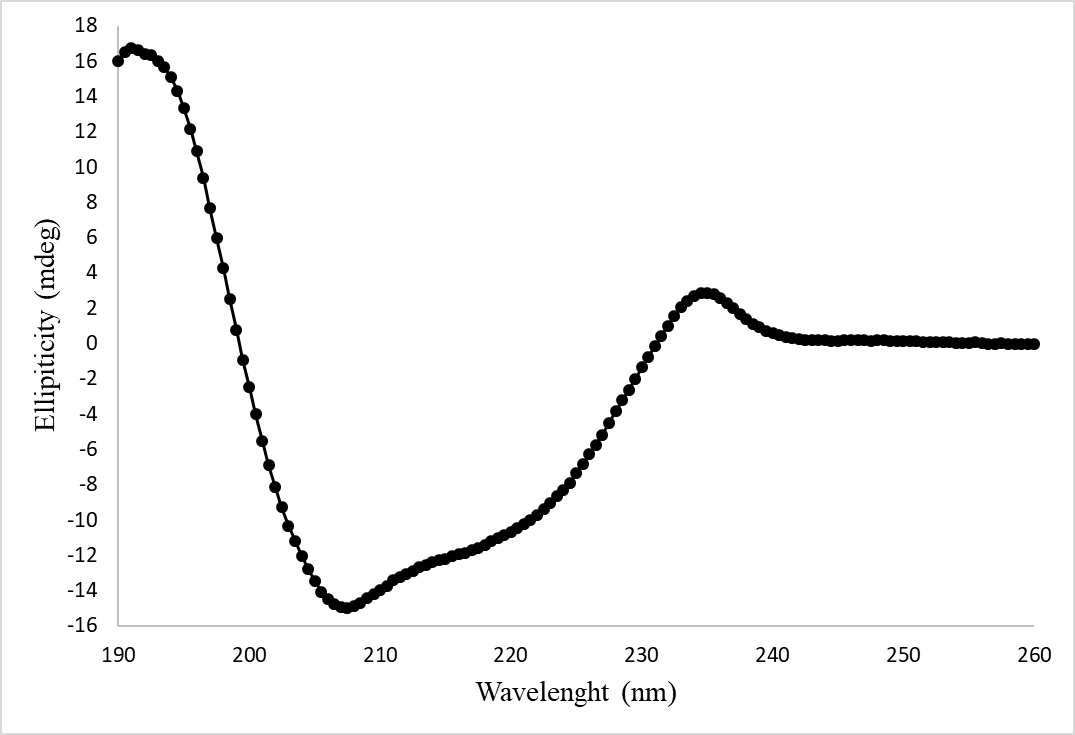


Figure S.5. Circular dichroism spectrum of MpPR-1i is consistent with spectra expected from an alpha-beta-alpha structure like the CAP proteins

**S.6) Thin Layer Chromatography (TLC) analysis of MpPR-1i delipidation**


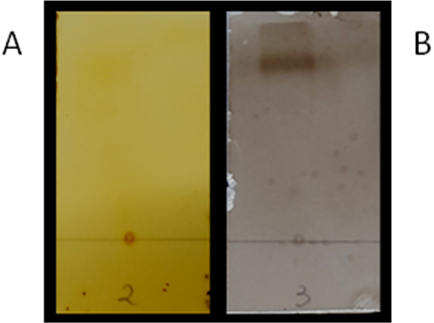


Figure S.6. Thin Layer Chromatography (TLC) analysis of MpPR-1i delipidation. Solvents used were the following: cyclohexane: ethyl acetate (4:1) for unsaturated neutral lipids (plate labeled 2) and chloroform: methanol: water (75:25:2.5) for saturated neutral lipids (plate labeled 3). The band in plate 3 indicates the presence of a saturated neutral lipid.

**S.7) Anomalous scattering and phasing**


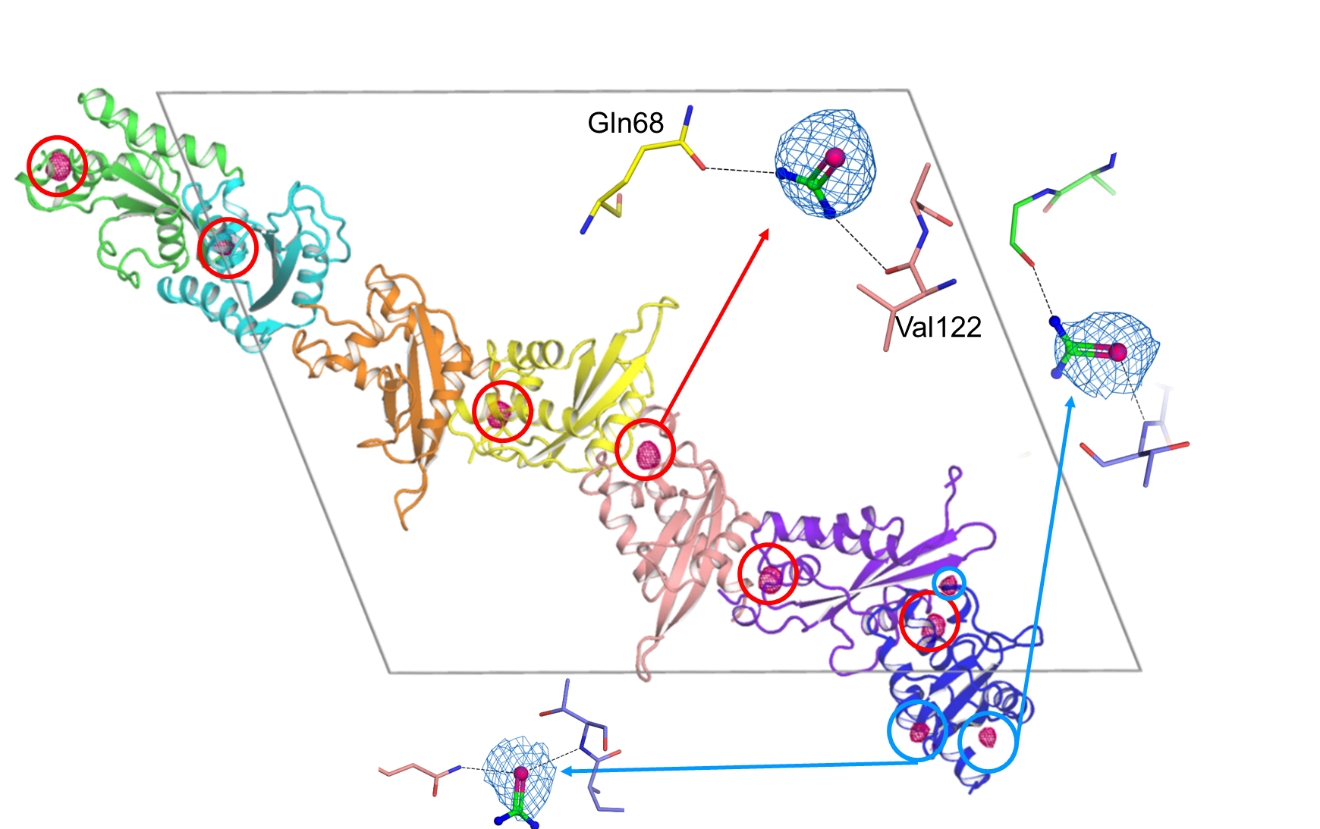


Figure S.7. Se atom binding sites and SeUrea binding details. The anomalous difference map at 4σ level is shown as red mesh. The 2Fo-Fc map of SeUrea molecules in the binding sites is shown as blue mesh at 1σ level. There are nine Se sites with six of them at the interface of adjacent monomers, highlighted in red circles.


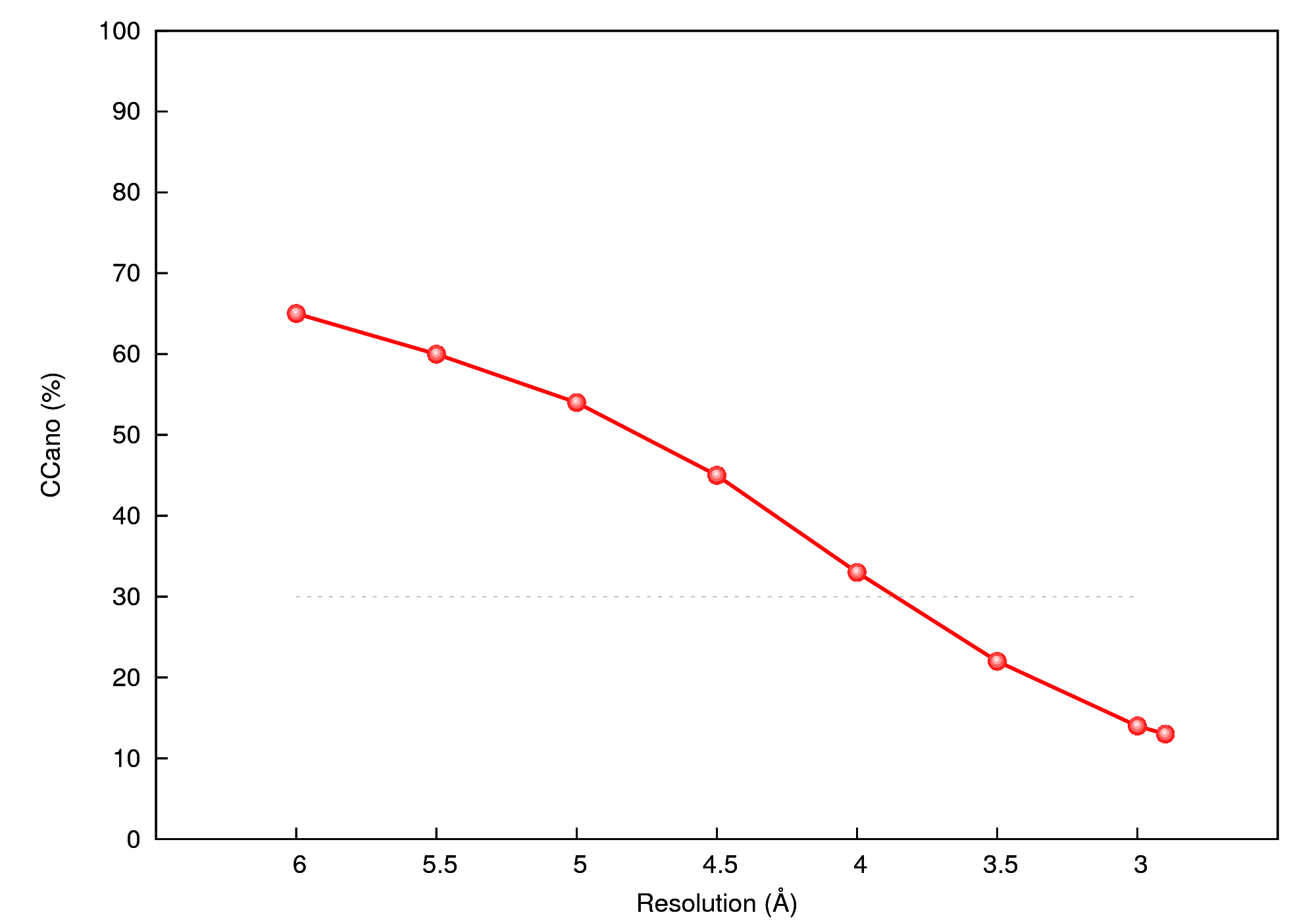


Figure S.8. Correlation coefficient for anomalous data set (CC_ano_) at different resolution.
